# Effects of Exercise-Based Interventions on Depressive Symptoms in Adults with Lung Cancer: A Systematic Review and Meta-Analysis of Randomized Controlled Trials

**DOI:** 10.3390/medicina62091805

**Published:** 2026-09-19

**Authors:** Mesut Süleymanoğulları, Zarife Pancar

**Affiliations:** 1 Department of Physical Education and Sports Teaching, Faculty of Sport Sciences, Ağrı İbrahim Çeçen University, Ağrı 04100, Türkiye; msuleymanogullari@agri.edu.tr; 2 Department of Physical Education and Sports, Faculty of Sports Science, Gaziantep University, Gaziantep 27350, Türkiye

**Keywords:** lung cancer, depression, exercise oncology, psycho-oncology, randomized controlled trials, meta-analysis

## Abstract

*Background and Objectives*: Exercise is increasingly used in supportive care for lung cancer, but its effects on depressive symptoms remain uncertain. This systematic review and meta-analysis synthesized randomized evidence. *Materials and Methods*: Four electronic databases were searched for reports published from January 2000 to July 2026. Eligible studies enrolled adults with lung cancer and compared a structured exercise-based intervention with usual care or an eligible active rehabilitation comparator. One depression result per independent trial was selected using documented rules for assessment time, outcome format, and multiple intervention arms. A random-effects model used REML with Hartung–Knapp inference. *Results*: Seventeen studies met the eligibility criteria; 15 independent trials (1041 participants) contributed to the primary synthesis. The pooled effect favored exercise (Hedges’ g = −0.75, 95% CI −1.18 to −0.32; *p* = 0.002), but heterogeneity was considerable (τ^2^ = 0.4582; τ = 0.6769; I^2^ = 87.1%; H^2^ = 7.76; Q(14) = 74.11, *p* < 0.001) and the 95% prediction interval crossed the null (−2.27 to 0.76). The direction remained favorable in all sensitivity analyses, although exclusion of two influential studies attenuated the pooled effect to −0.49 and reduced I^2^ to approximately 0%. Exploratory subgroup and study-level moderator analyses did not identify a stable explanation for heterogeneity. Certainty was very low after downgrading for risk of bias, inconsistency, and indirectness. *Conclusions*: Exercise-based interventions may reduce depressive symptom scores in some adults with lung cancer, but confidence in the magnitude and transferability of the average effect is limited. The evidence does not establish efficacy for major depressive disorder or identify an optimal modality, dose, or delivery format.

## 1. Introduction

Lung cancer was the most frequently diagnosed cancer worldwide in 2022 and remained the leading cause of cancer-related death, accounting for approximately 2.5 million new cases and 1.8 million deaths [1]. For many patients, however, the burden of the disease extends beyond tumor-related symptoms and treatment adverse effects. Psychological distress may arise at diagnosis and persist during treatment, recovery, or advanced disease.

Longitudinal evidence from patients with lung cancer indicates that depressive symptoms may develop or persist after diagnosis and are associated with poorer quality of life, greater symptom burden, lower perceived social support, and shorter survival [2]. Depressive symptoms are also common among adults with cancer, although prevalence estimates vary according to the population studied and the method of assessment [3]. These symptoms rarely have a single cause. Dyspnea, fatigue, pain, loss of physical function, treatment-related adverse effects, social isolation, and lung cancer-related stigma may all contribute [2,4]. Oncology guidelines therefore recommend regular assessment and management according to symptom severity and individual clinical needs [4,5]. Delivering this care can nevertheless be difficult, particularly when depressive and cancer-related somatic symptoms overlap, access to psycho-oncology services is limited, or patients already face a substantial treatment burden.

Exercise is relevant in this context because it may address physical functioning and psychological well-being within the same supportive-care program. International guidelines recommend appropriately prescribed aerobic and resistance exercise during and after cancer treatment when it is clinically suitable [6,7]. Meta-analyses conducted in cancer survivors have reported reductions in depressive symptoms, while evidence from the broader depression literature also supports a potential role for exercise [8,9,10]. These findings cannot be transferred directly to people with lung cancer. Respiratory limitation, cachexia, comorbidities, performance status, and anticancer treatment may affect both the ability to participate in exercise and the response to an exercise program.

This distinction is important because trials conducted in lung cancer represent neither a single clinical population nor a uniform intervention. Participants have been recruited before and after surgery, during chemotherapy or radiotherapy, with advanced disease, and in survivorship settings. Most trials did not require clinically significant depressive symptoms at enrollment, and depression was commonly assessed as a secondary outcome. The evidence may therefore reflect both improvement in existing symptoms and prevention of symptom-worsening and does not establish treatment efficacy for major depressive disorder. Interventions have ranged from supervised aerobic and resistance training to walking, breathing-based and mind–body exercise, home rehabilitation, and digitally supported multicomponent programs. Comparator conditions have ranged from usual care to education, self-management, and active rehabilitation. Intervention intensity, depression instruments, and assessment time points have also differed. Previous lung cancer-specific reviews have reported potentially favorable psychological outcomes but have consistently described a small and clinically heterogeneous evidence base [11,12,13,14]. Placing these studies on a common statistical scale may allow an average effect to be estimated, but it does not make their populations, interventions, comparator conditions, or clinical settings interchangeable.

Against this background, we conducted a systematic review and meta-analysis of randomized controlled trials evaluating structured exercise-based interventions in adults with lung cancer. The primary objective was to estimate the average effect on post-intervention depressive symptoms and quantify variation across trials. We also examined the stability of the pooled estimate and explored whether exercise category, depression instrument, intervention duration, and selected study-level participant characteristics contributed to between-study heterogeneity. Because the evidence base was small and clinically diverse, moderator analyses were treated as exploratory and hypothesis-generating.

## 2. Materials and Methods

### 2.1. Registration

**Figure 1:**
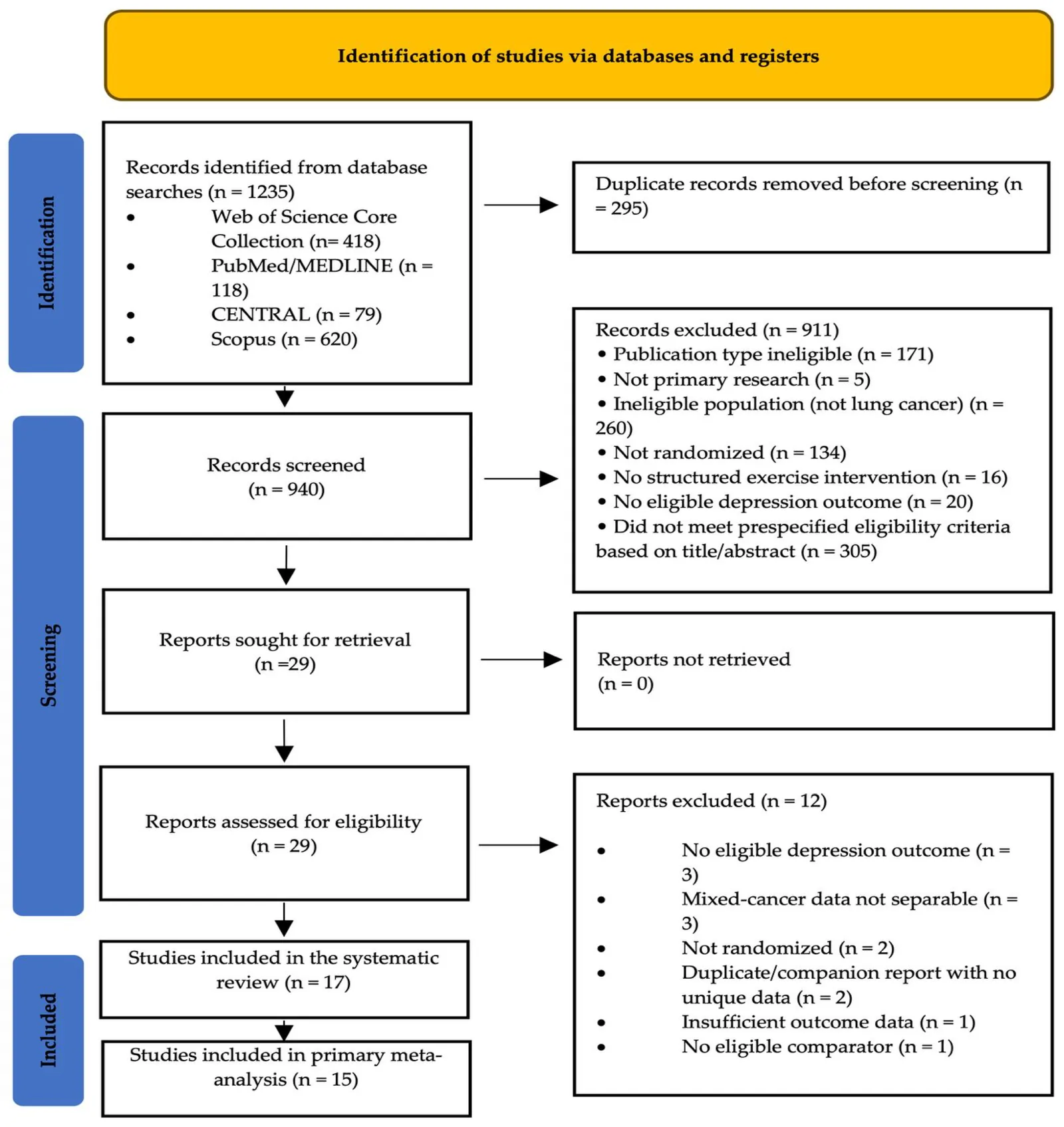
PRISMA 2020 flow diagram of the study selection process.

This systematic review and meta-analysis was conducted in accordance with the Preferred Reporting Items for Systematic Reviews and Meta-Analyses (PRISMA) 2020 statement [15], and the study selection process is shown in Figure 1. As this study was a systematic review and meta-analysis based exclusively on data from previously published studies, no new human participants were recruited, no interventions were administered by the authors, and no identifiable individual-level data were collected. Therefore, institutional ethics committee approval and informed consent were not required for the present study. Ethical approval and informed consent procedures for the individual studies included in this review were the responsibility of the respective original investigators and institutions.

The review was registered in the Open Science Framework Registries on 17 July 2026 (registration version 1; https://osf.io/vuc87/ (accessed on 17 July 2026)). Database searching, screening, data extraction, risk-of-bias assessment, and statistical analysis had been completed before registration. Because registration was retrospective, the OSF record did not function as a prospective protocol.

Analytical differences between registration version 1 and the final report were limited to analyses that could not be implemented reliably or were added for exploratory purposes. Stage- and treatment-based subgroup analyses were not performed because the available categories were sparse, overlapping, and incompletely reported. Fail-safe N was not reported because it was considered to have limited interpretability in the presence of substantial between-study heterogeneity. Additional study-level moderator analyses were reported as exploratory and hypothesis-generating.

The registration record stated that screening, data extraction, and risk-of-bias assessment were conducted independently by two reviewers (M.S. and Z.P.). Title-and-abstract screening and full-text assessment were conducted independently by the same reviewers, as described in Section 2.4. Data extraction was performed by one reviewer (M.S.), who checked all entries against the source reports. Risk-of-bias assessment was performed by two reviewers (M.S. and Z.P.). Independent duplicate data extraction was not performed. The procedures implemented at each stage of the review are reported in Section 2.4, Section 2.5, Section 2.6 and Section 2.7.

### 2.2. Eligibility Criteria

Eligibility was defined using PICOS (Population, Intervention, Comparator, Outcomes, and Study Design). Eligible participants were adults (≥18 years) with a confirmed diagnosis of lung cancer, irrespective of histological subtype, disease stage, treatment phase, or clinical setting. Eligible interventions comprised structured exercise or exercise-based rehabilitation delivered either alone or as a substantive component of a multicomponent rehabilitation program. These included aerobic, resistance, combined, pulmonary rehabilitation, breathing-based, mind–body, walking, digitally delivered, and other structured exercise interventions. Exercise was considered a substantive intervention component when it was scheduled, repeated, and prescribed in terms of content and dose rather than limited to general physical-activity advice. Eligible comparators included usual care; education, attention, or self-management controls; non-exercise control conditions; and active rehabilitation comparators when the intervention arm delivered an additional, more intensive, or substantively different structured exercise or rehabilitation program. Eligible outcomes were depressive symptoms assessed using validated instruments or separate extractable depression subscales.

Eligible studies were parallel-group randomized controlled trials published as full-text, peer-reviewed articles in English between January 2000 and July 2026. Studies were excluded if participants did not have lung cancer, treatment allocation was not randomized, the intervention did not include structured exercise, depression outcomes were unavailable or not separately extractable, or the publication was not an original peer-reviewed research article. Multi-arm randomized trials were eligible and were handled according to the documented data-synthesis procedures.

### 2.3. Search Strategy

Electronic searches were conducted in PubMed/MEDLINE, Scopus, Web of Science Core Collection, and the Cochrane Central Register of Controlled Trials (CENTRAL) on 12 July 2026. Searches were limited to English-language records published from 1 January 2000 to 12 July 2026. The searches retrieved 118 records from PubMed/MEDLINE, 620 from Scopus, 418 from Web of Science Core Collection, and 79 from CENTRAL, resulting in a total of 1235 records before deduplication. Supplementary Materials Table S1 provides the complete database-specific search strategies used, including controlled vocabulary, field restrictions, applied limits, search dates, and the number of records retrieved from each database before deduplication.

### 2.4. Study Selection

Database records were standardized and deduplicated using DOI, PMID, and compatible bibliographic fields. Two reviewers (M.S. and Z.P.) independently screened titles and abstracts against the eligibility criteria. Reports considered potentially eligible by either reviewer proceeded to full-text assessment. The same reviewers independently assessed the full-text reports, recorded one principal reason for each exclusion, and resolved disagreements through discussion and consensus. Multiple reports originating from the same randomized trial were linked using a unique study identifier to ensure that each trial contributed only one independent primary dataset to the quantitative synthesis.

### 2.5. Data Extraction

One reviewer (M.S.) extracted study characteristics and outcome data into structured forms and checked all entries against the corresponding source reports during construction of the analysis-ready dataset. A completed independent duplicate data-extraction procedure was not performed. Randomized, baseline-demographic, analyzed, and depression-outcome populations were recorded separately. Extracted fields included participant characteristics, intervention and comparator characteristics, depression instrument, assessment time, analysis population, summary statistics, attrition, adherence, and funding information when reported.

All eligible arm-level depression results were retained. Published endpoint means and SDs were preferred, followed by validly derived endpoints, adjusted endpoints with compatible SE-to-SD conversion, and change scores when no compatible endpoint was available. Median conversions used the Luo mean estimator and the Shi or Wan SD estimator, as appropriate to the reported data [16,17,18]. SD was calculated as SE × √N only when the corresponding group-specific sample size was available. One independent result was then selected from each trial. Compatible eligible intervention arms sharing one control group were combined using Cochrane continuous-outcome formulas, with the control group included only once; otherwise, the contrast that most directly isolated the additional structured exercise component was selected [19].

### 2.6. Outcome Definition

The selected outcome was a validated depression scale or separately extractable depression subscale. No quantitative trial reported more than one competing eligible depression instrument, so no instrument-priority choice was required. The assessment closest to intervention completion was selected, preferring immediate post-intervention data. Published unadjusted endpoints were preferred; valid derived or adjusted endpoints followed, and change scores were used only when no compatible endpoint was available. Adjusted and unadjusted estimates from the same participants were not entered together. Longer follow-up results were retained separately. Higher scores consistently indicated more severe depressive symptoms across all included instruments; therefore, negative standardized effect sizes favored exercise. Longer-term follow-up outcomes were retained for qualitative reporting but were not included in the primary meta-analysis. Studies reporting only the total Hospital Anxiety and Depression Scale (HADS) score, without a separately extractable Hospital Anxiety and Depression Scale–Depression (HADS-D) subscale, were excluded from the quantitative synthesis but retained in the qualitative review.

### 2.7. Risk of Bias and Certainty of Evidence

Risk of bias for the selected depression result was assessed by two reviewers (M.S. and Z.P.) using the Cochrane RoB 2 tool [20]. Disagreements were resolved through discussion and consensus. Signaling-question responses, supporting evidence, domain-level judgments, and overall judgments were recorded for each included study. GRADE was applied to the primary outcome, with each downgrade linked to the corresponding body of evidence [21].

### 2.8. Statistical Analysis

All analyses were conducted in R (version 4.4.1; R Foundation for Statistical Computing, Vienna, Austria) using the metafor package [22]. For each independent comparison, Hedges’ g and its sampling variance were calculated with the intervention group entered first and the comparator group second. Because higher scores indicated more severe depressive symptoms, negative effect estimates favored the intervention. Unusually large effect estimates were checked against the source data, and no study was excluded solely because of effect magnitude. A random-effects model was used because the true intervention effects were expected to vary across studies. The primary meta-analysis used restricted maximum likelihood (REML) estimation with Hartung–Knapp inference [23]. Statistical significance was defined using a two-sided α of 0.05. The pooled effect estimate, 95% confidence interval, 95% prediction interval, τ^2^, τ, I^2^, H^2^, and Cochran’s Q statistic were reported. Following Higgins et al. [24], I^2^ was interpreted as an index of between-study inconsistency rather than methodological quality. Robustness analyses examined the exclusion of the high-risk study, influential studies, studies requiring converted or summary statistics, studies requiring the combination of multiple intervention arms, and studies without directly reported endpoint means, SDs, and group sizes. Leave-one-out analyses and alternative random-effects model specifications were examined separately. Subgroup analyses and univariable study-level meta-regressions were exploratory, were not adjusted for multiple testing, and were interpreted as hypothesis-generating. Small-study effects were assessed using funnel-plot inspection, Egger’s regression test, Begg’s rank-correlation test, and trim-and-fill analysis; these findings were interpreted cautiously because the number of trials was small and between-study heterogeneity was substantial [25,26,27]. An additional sensitivity analysis restricted the synthesis to exercise-dominant comparisons. Interventions were classified as exercise-dominant when their principal therapeutic content comprised scheduled, repeated, and dose-specified aerobic, resistance, walking, breathing, mind–body, or pulmonary rehabilitation exercise; digital delivery or monitoring alone did not result in exclusion. Programs in which education, psychological support, nutrition, or general self-management constituted coequal therapeutic components were exclude. 

## 3. Results

### 3.1. Study Selection Results

Twenty-nine full-text reports were assessed for eligibility. Seventeen unique randomized studies met the eligibility criteria, whereas 12 reports were excluded (Figure 1). Fifteen independent trials contributed one depression effect each to the primary quantitative synthesis. Liu et al. (2024) [28] was retained in the qualitative synthesis because the reported distribution could not be converted using the applicable conversion methods. Tenconi et al. [29] was retained in the qualitative synthesis because only the total HADS score, without a separately extractable HADS-D subscale, was reported.

### 3.2. Study Characteristics

The 17 eligible studies evaluated conventional exercise or pulmonary rehabilitation, mind–body or breathing-based exercise, and digital, behavioral, or multicomponent exercise rehabilitation across perioperative, active-treatment, advanced-disease, and survivorship settings (Table 1). Table 1 distinguishes randomized, baseline-demographic, analyzed, and selected depression-analysis populations because these denominators differed in several reports. The 15 quantitative trials contributed 543 intervention and 498 comparator participants (total N = 1041). Cheung et al. [30] and Wang et al. [31] were multi-arm trials. The loaded-deep-breathing arm was compared with routine care, and the PERMA-containing arm was excluded from this analysis. For quantitative synthesis, eligible intervention arms were combined within each study and the shared comparator group was counted only once, thereby retaining one statistically independent primary comparison per trial. Comparator conditions ranged from usual care, education or attention control, and self-management to active rehabilitation. In trials using pulmonary rehabilitation or chest physiotherapy as active comparators, the effect estimates represent comparative or incremental effects relative to active rehabilitation rather than exercise versus no exercise.

### 3.3. Risk of Bias

**Figure 2:**
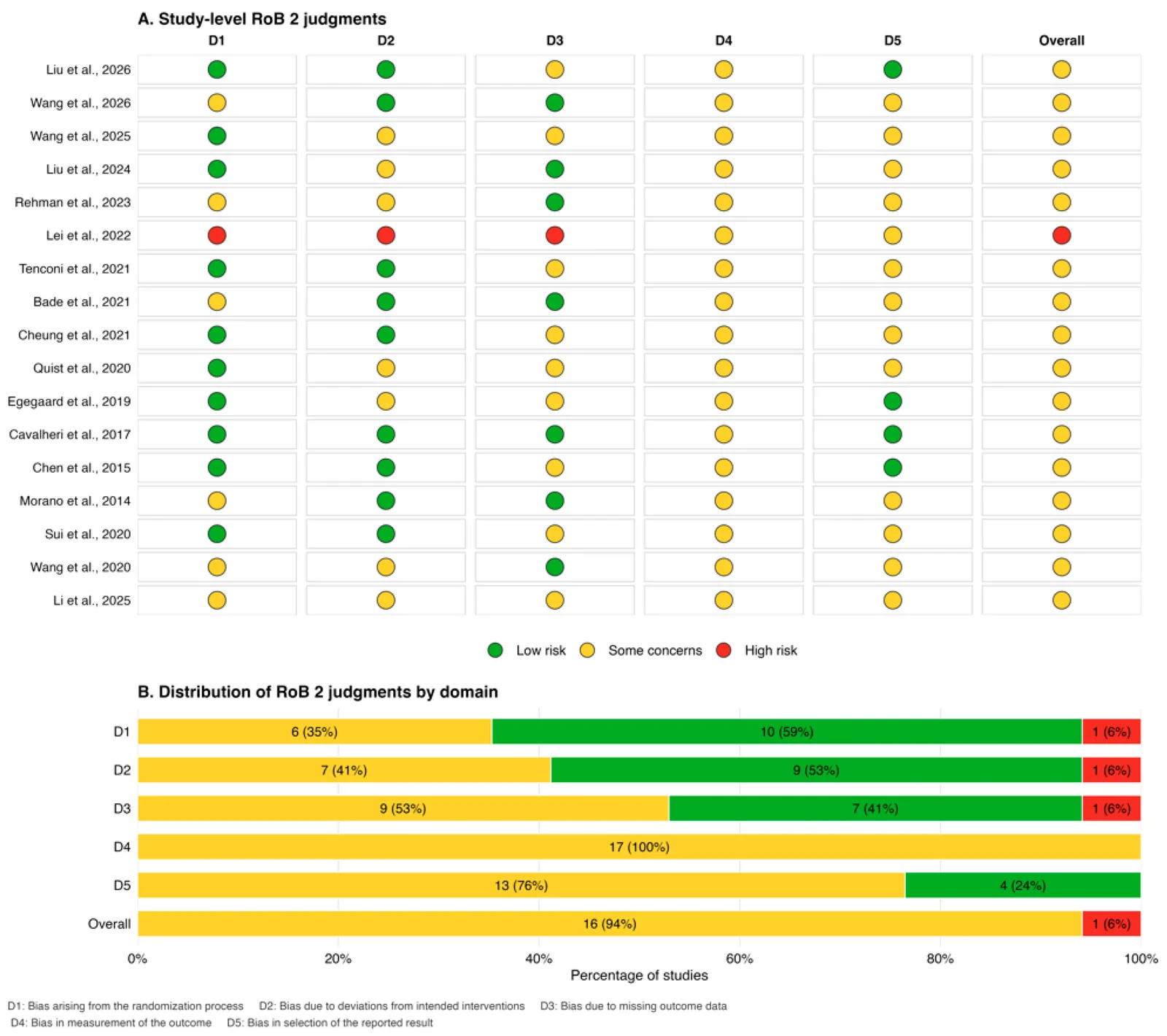
Risk-of-bias assessment using the Cochrane RoB 2 tool [28,29,30,31,32,33,34,35,36,37,38,39,40,41,42,43,44].

All 17 studies underwent result-specific RoB 2 assessment (Figure 2). Concerns most often involved self-reported depression in an unblinded comparison, missing outcome data, or selection of the reported result.

### 3.4. Primary Meta-Analysis

**Figure 3:**
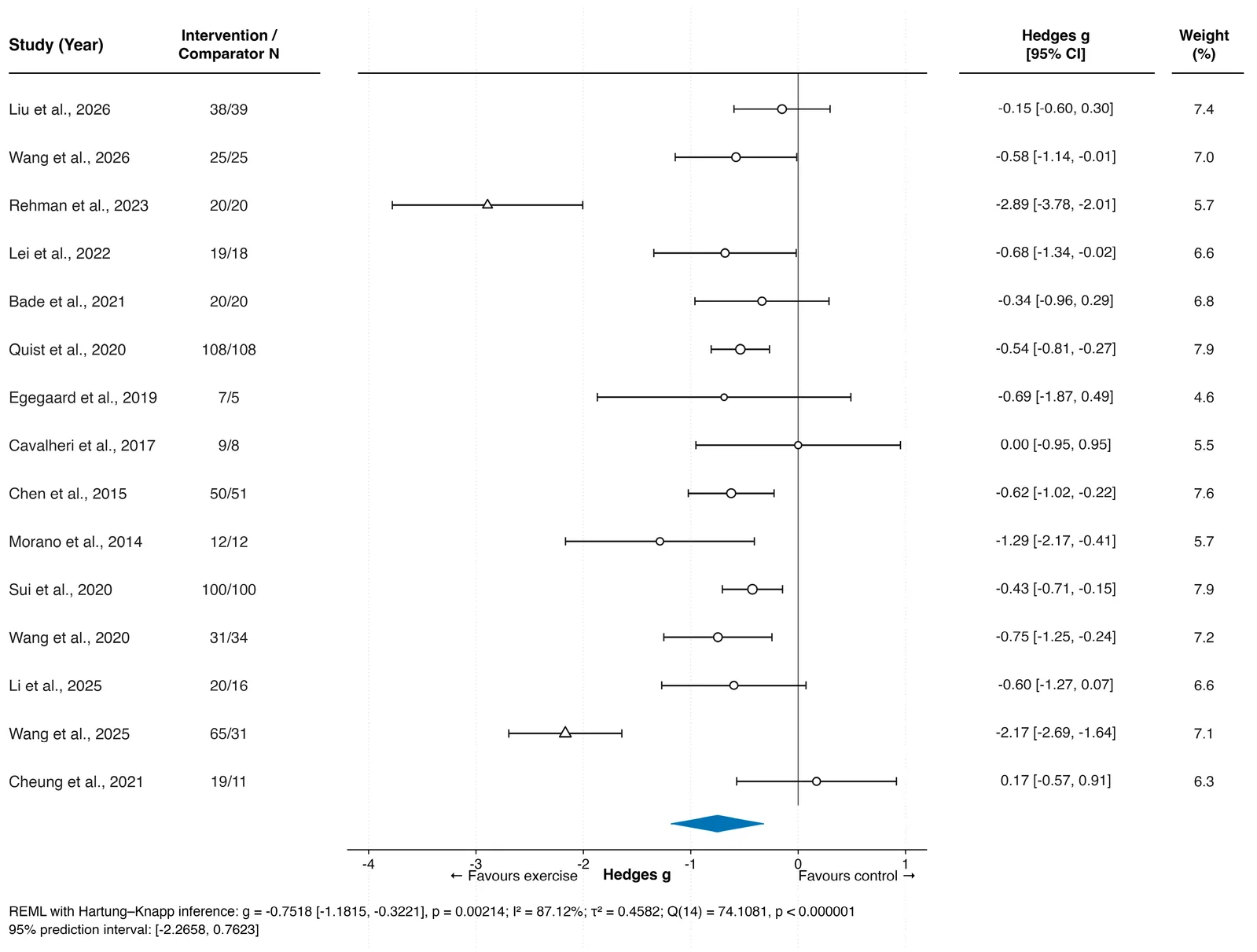
Primary random-effects meta-analysis [30,31,32,33,34,35,36,37,38,39,40,41,42,43,44]. Notes: Triangles indicate studies identified as influential in the influence diagnostics; circles indicate the remaining studies.

The primary synthesis included 15 independent trials and 1041 participants (Figure 3). Negative values favored exercise. REML with Hartung–Knapp inference yielded Hedges’ g = −0.7518 (95% CI −1.1815 to −0.3221; t(14) = −3.7525; *p* = 0.00214). Heterogeneity was substantial: τ^2^ = 0.4582, τ = 0.6769, I^2^ = 87.12%, H^2^ = 7.7619, and Q(14) = 74.1081 (*p* = 3.45 × 10^−10^). The 95% prediction interval was −2.2658 to 0.7623. Influence diagnostics identified Rehman et al. [34] as influential and Wang et al. [31] as potentially influential.

### 3.5. Sensitivity Analyses

**Figure 4:**
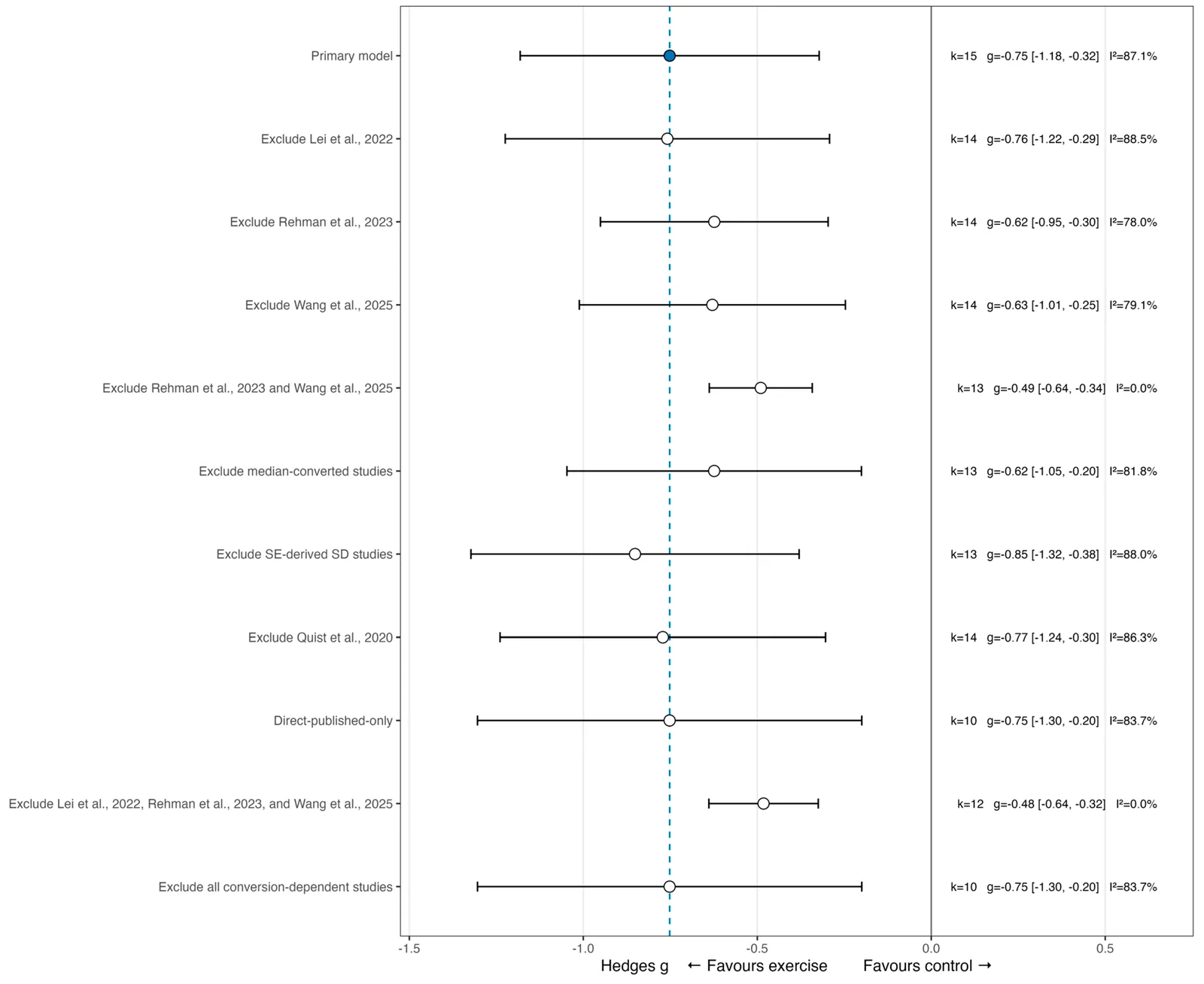
Sensitivity analyses of the primary pooled effect [31,34,35,37]. Notes: Circles represent pooled Hedges’ g estimates and horizontal lines represent 95% confidence intervals. The blue dashed line indicates the primary pooled estimate. Abbreviations: CI, confidence interval; *k*, number of trials; I^2^, percentage of variability attributable to between-study inconsistency; SD, standard deviation; SE, standard error.

Sensitivity analyses retained the same effect-size coding and REML/Hartung–Knapp method used in the primary analysis (Figure 4). Excluding Lei et al. [35], the study judged to be at high risk of bias, yielded g = −0.7584 (95% CI, −1.2244 to −0.2923; I^2^ = 88.48%; k = 14). Excluding Rehman et al. [34] yielded g = −0.6235 (95% CI, −0.9507 to −0.2964; I^2^ = 77.95%), whereas excluding Wang et al. [31] yielded g = −0.6291 (95% CI, −1.0115 to −0.2468; I^2^ = 79.14%). Joint exclusion of Rehman et al. [34] and Wang et al. [31] attenuated the pooled effect to g = −0.4900 (95% CI, −0.6380 to −0.3419) and reduced heterogeneity to approximately zero (I^2^ ≈ 0%; k = 13). Source-data verification for both influential trials confirmed the extracted group sizes, HADS-D scale units, and baseline and post-intervention summary statistics; no transcription or unit errors or other documented reasons for exclusion were identified. Both trials were therefore retained in the primary synthesis and examined in influence and sensitivity analyses. The analysis restricted to trials with directly reported endpoint means, SDs, and group sizes included 10 trials and yielded g = −0.7520 (95% CI, −1.3042 to −0.1997; I^2^ = 83.70%). Other conversion- and derivation-based sensitivity analyses are presented in Figure 4.

Excluding the two median-converted trials yielded g = −0.6238 (95% CI, −1.0471 to −0.2006; I^2^ = 81.78%; k = 13), whereas excluding trials with SE-derived SDs yielded g = −0.8514 (95% CI, −1.3230 to −0.3797; I^2^ = 88.03%; k = 13). Exclusion of the trial requiring the documented equal-allocation group-size rule yielded g = −0.7716 (95% CI, −1.2393 to −0.3038; I^2^ = 86.34%; k = 14). Thus, the favorable average direction was retained across data-transformation sensitivity analyses, although substantial heterogeneity generally persisted.

In the exercise-dominant sensitivity analysis, the HIM program [33] and WeChat program [38] were excluded, and the loaded-deep-breathing-only arm from Wang et al. [31] was compared with routine care. Thirteen independent comparisons involving 757 participants yielded g = −0.7703 (95% CI −1.2525 to −0.2880; *p* = 0.0045; τ^2^ = 0.4766; I^2^ = 85.35%). The 95% prediction interval crossed the null (−2.3498 to 0.8093). Restricting the synthesis to exercise-dominant comparisons therefore retained a favorable average direction but did not resolve heterogeneity or uncertainty in transferability.

**Figure 5:**
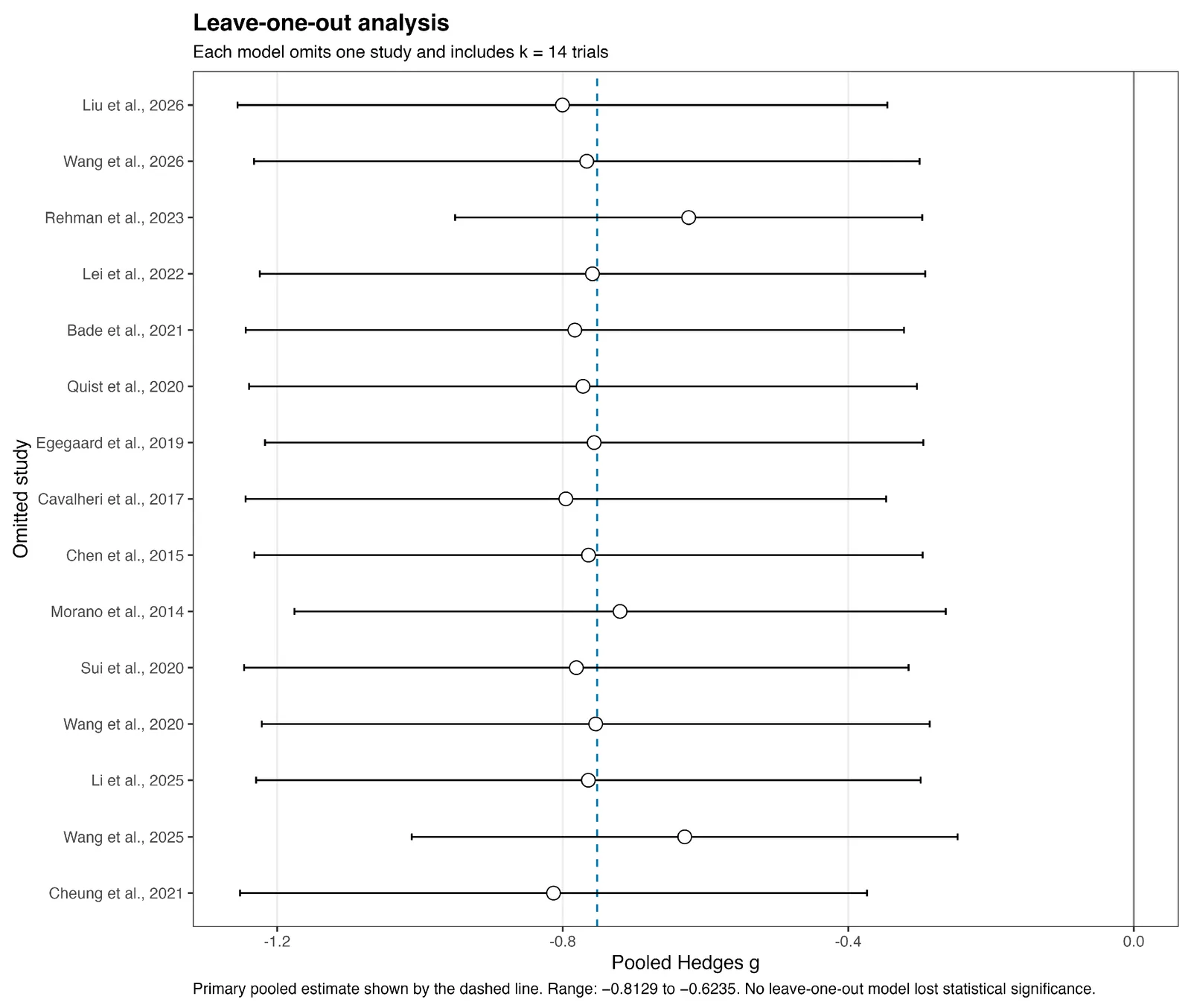
Leave-one-out sensitivity analysis [30,31,32,33,34,35,36,37,38,39,40,41,42,43,44]. Notes: Each row presents the pooled Hedges’ g estimate after omission of the indicated study; all models included 14 trials. Circles represent pooled estimates and horizontal lines represent 95% confidence intervals. The blue dashed line indicates the primary pooled estimate. Negative values favor exercise. No leave-one-out model changed the favorable direction or statistical significance of the pooled estimate. Abbreviations: CI, confidence interval; k, number of trials.

Leave-one-out analyses yielded pooled estimates ranging from g = −0.8129 to −0.6235 (Figure 5). Omission of any single trial changed neither the favorable direction nor the statistical significance of the pooled estimate. Nevertheless, stability of direction should not be interpreted as stability of effect magnitude or heterogeneity. In particular, the joint exclusion of Rehman et al. [34] and Wang et al. [31] attenuated the pooled effect from g = −0.75 to −0.49 and reduced I^2^ from 87.12% to approximately 0%. The magnitude and heterogeneity of the primary estimate were therefore sensitive to a small number of clinically and analytically distinctive trials.

### 3.6. Exercise Subgroup Analysis

**Figure 6:**
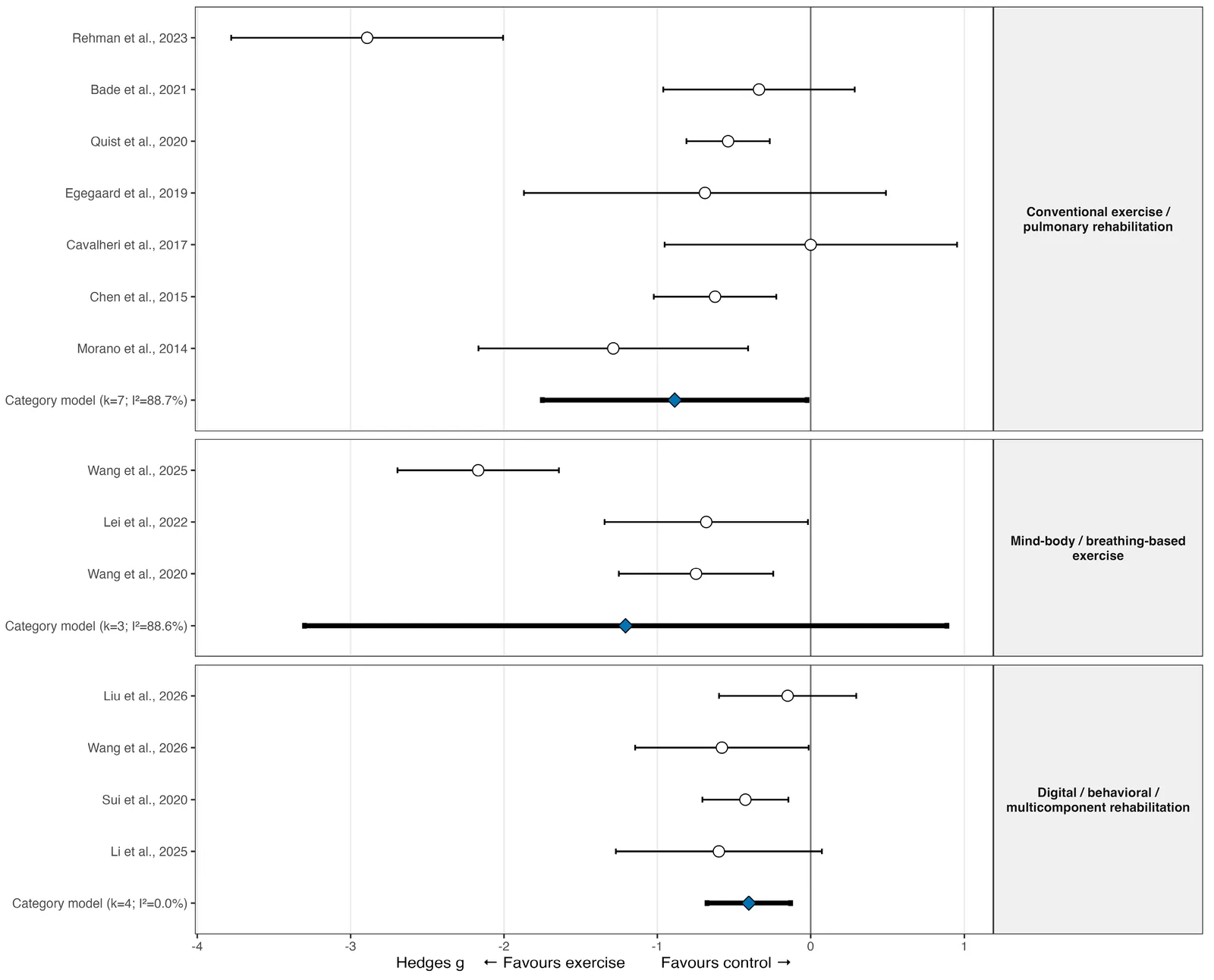
Exploratory exercise-category subgroup analysis [31,32,33,34,35,36,37,38,39,40,41,42,43,44]. Notes: Circles represent study-specific Hedges’ g estimates and horizontal lines represent 95% confidence intervals. Diamonds represent pooled category estimates. Negative values favor exercise. Cheung et al. [30] remained in the primary meta-analysis but was excluded from this subgroup analysis because its combined intervention group included both aerobic exercise and Tai Chi. Abbreviations: CI, confidence interval; k, number of trials; I^2^, percentage of variability attributable to between-study inconsistency.

The exploratory exercise-category subgroup analysis included 14 trials. Cheung et al. [30] was excluded from this analysis because its combined intervention group comprised both aerobic exercise and Tai Chi and therefore could not be assigned exclusively to one exercise category; the study remained included in the primary meta-analysis (Figure 6). Seven trials classified as conventional exercise or pulmonary rehabilitation [34,36,37,40,41,42,43] yielded a pooled Hedges’ g of −0.8866 (95% CI, −1.7498 to −0.0234; *p* = 0.0457; τ^2^ = 0.6807; I^2^ = 88.70%). Three trials classified as mind–body or breathing-based exercise [31,35,39] yielded g = −1.2074 (95% CI, −3.3008 to 0.8860; *p* = 0.1312; τ^2^ = 0.6318; I^2^ = 88.58%). Four trials classified as digital, behavioral, or multicomponent rehabilitation [32,33,38,44] yielded g = −0.4034 (95% CI, −0.6759 to −0.1309; *p* = 0.0181; τ^2^ = 0; I^2^ = 0.00%). The omnibus test showed no statistically significant differences between exercise categories (QM (2) = 0.9990; *p* = 0.3994). Exercise category explained little of the estimated between-study heterogeneity (pseudo-R^2^ = 2.91%; residual τ^2^ = 0.4257; residual I^2^ = 85.20%). These exploratory between-study comparisons do not provide evidence that any exercise category is superior to another.

### 3.7. Depression-Instrument Subgroup Analysis

**Figure 7:**
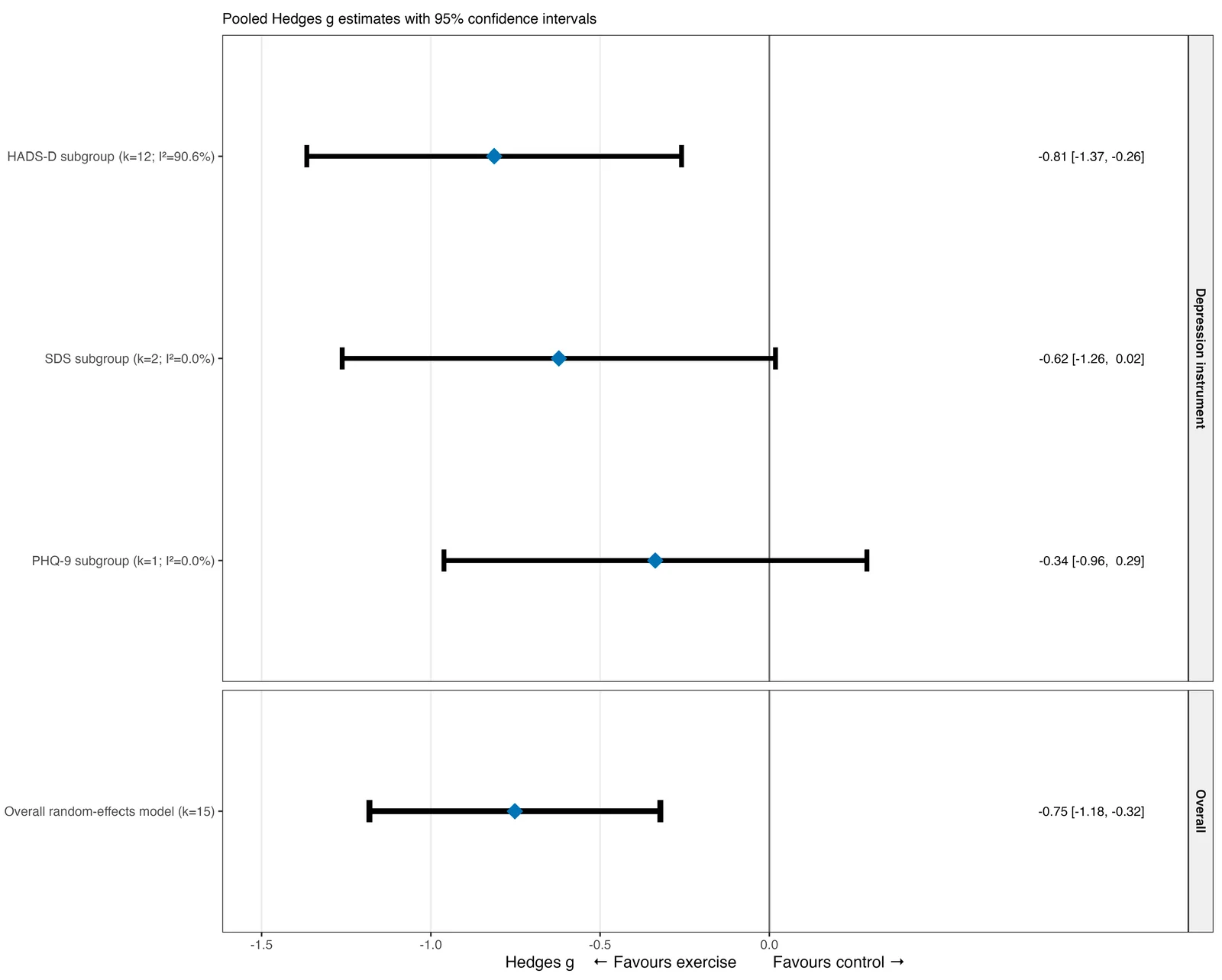
Exploratory subgroup analysis according to depression instrument. Notes: Diamonds represent subgroup and overall estimates with 95% confidence intervals. Negative Hedges’ g values favor exercise. The PHQ-9 result represents a single-study descriptive estimate. Instrument-specific estimates should be interpreted cautiously because the SDS and PHQ-9 categories contained few trials.

The exploratory depression-instrument subgroup analysis (Figure 7) included all 15 independent trials. Twelve trials used the Hospital Anxiety and Depression Scale–Depression subscale (HADS-D), two used the Self-Rating Depression Scale (SDS), and one used the Patient Health Questionnaire-9 (PHQ-9). The pooled effect estimate for the HADS-D subgroup was g = −0.8129 (95% CI, −1.3665 to −0.2594; *p* = 0.00798; k = 12), whereas the estimate for the SDS subgroup was g = −0.6219 (95% CI, −1.2619 to 0.0181; *p* = 0.05145; k = 2). The single PHQ-9 trial yielded g = −0.3371 (95% CI, −0.9613 to 0.2871; *p* = 0.28978) and was presented descriptively rather than as a pooled subgroup estimate. The omnibus test showed no statistically significant differences between depression instruments (QM(2) = 0.1796; *p* = 0.8378). Residual heterogeneity remained substantial (τ^2^ = 0.5461; I^2^ = 89.29%), and the instrument moderator explained none of the estimated between-study heterogeneity (pseudo-R^2^ = 0.00%). Although the HADS-D subgroup estimate excluded the null, the nonsignificant omnibus test provides no evidence that intervention effects differed according to the depression instrument used. Because the SDS subgroup contained only two trials and PHQ-9 was represented by a single trial, these instrument-specific findings should be interpreted cautiously.

### 3.8. Small-Study-Effect Diagnostics

**Figure 8:**
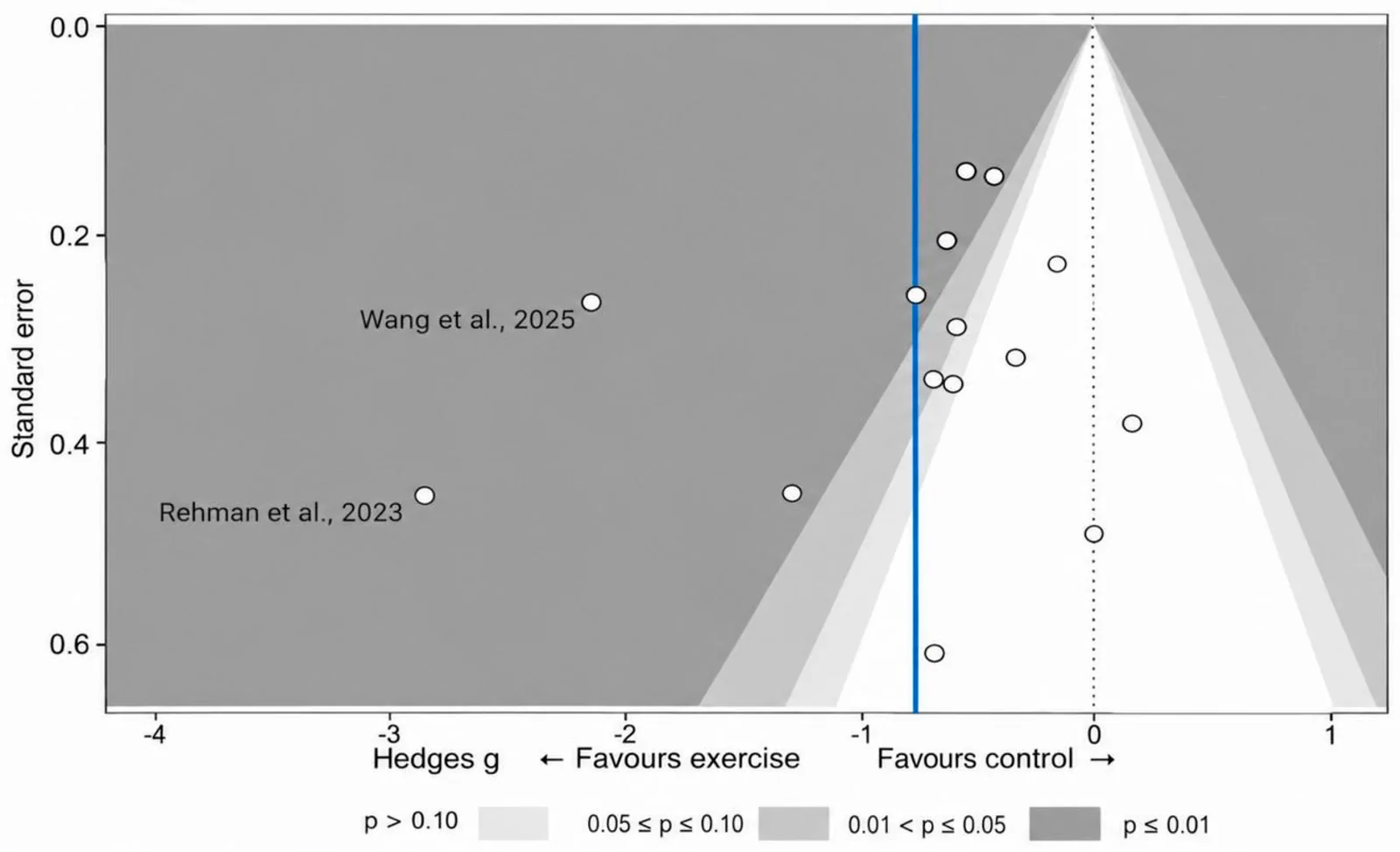
Contour-enhanced funnel plot of the 15 trials included in the primary meta-analysis [31,34]. Notes: Circles represent individual trial estimates plotted against their standard errors. The dotted vertical line indicates the null effect, and the blue vertical line indicates the primary pooled estimate. Shaded contours represent two-sided statistical-significance regions around the null. Negative Hedges’ g values favor exercise. This diagnostic does not establish the presence or absence of publication bias. Abbreviation: SE, standard error.

Small-study-effect diagnostics included all 15 trials contributing to the primary synthesis (Figure 8). The Egger-type regression did not indicate statistically significant funnel-plot asymmetry (coefficient = −1.1540, SE = 1.6925; t(13) = −0.6818; *p* = 0.5073), and the Begg–Mazumdar rank-correlation test was also nonsignificant (Kendall’s τ = −0.1619; *p* = 0.4351) [25,26,27]. Exploratory trim-and-fill analysis imputed no studies, and the pooled estimate remained unchanged (Hedges’ g = −0.75, 95% CI −1.18 to −0.32). The Egger and rank-correlation tests remained nonsignificant after excluding Rehman et al. [34], Wang et al. [31], or both trials. These diagnostics did not provide clear statistical evidence of small-study effects. Nevertheless, their power and specificity were limited by the small number of trials and substantial between-study heterogeneity; therefore, these findings cannot establish the absence of publication bias.

### 3.9. Meta-Regression

Study-level mean age was available for 14 trials. Each additional year of mean age was associated with a 0.0814-unit increase in Hedges’ g, corresponding to a smaller estimated benefit of exercise (95% CI, 0.0074 to 0.1554; *p* = 0.0338). Substantial residual heterogeneity remained (τ^2^ = 0.3343; I^2^ = 83.01%), and the association was sensitive to Rehman et al. [34]. This univariable ecological association was not adjusted for multiple testing and does not support patient-level treatment selection. Intervention duration was available for 13 trials and was not associated with effect magnitude (β = 0.0113 per week; 95% CI, −0.0219 to 0.0445; *p* = 0.4689).

Baseline depression means on the selected instrument were available for 12 quantitative trials. None of the 17 eligible studies required elevated depressive symptoms for enrollment. In an exploratory POMP-standardized study-level analysis, each 10-percentage-point increase in baseline depressive symptom burden was associated with a more negative effect estimate (β = −0.409; 95% CI, −0.633 to −0.186; *p* = 0.0022; k = 12). This association was sensitive to Rehman et al. [34]. Because these analyses were univariable, ecological, and unadjusted for multiple testing, they are presented as hypothesis-generating and do not establish patient-level effect modification.

### 3.10. GRADE Certainty

GRADE was applied to depressive symptoms assessed at the time point closest to intervention completion (Table 2). Randomized evidence started at high certainty and was downgraded by one level for risk of bias because no trial was judged to be at overall low risk and one was judged to be at high risk. Evidence was downgraded by one level for serious inconsistency because between-study heterogeneity was substantial and the 95% prediction interval crossed the null. Evidence was downgraded once more for serious indirectness because no trial required clinically elevated depressive symptoms for enrollment, depression was commonly a secondary outcome, several interventions were complex or multicomponent, comparators included both usual care and active rehabilitation, and clinical settings ranged from surgery to advanced disease and survivorship.

Imprecision was not downgraded separately. No validated cross-instrument minimal important difference was available; therefore, an absolute standardized effect of 0.20 was used as an interpretive small-effect threshold. The Hartung–Knapp confidence interval (g = −1.18 to −0.32) excluded both the null and effects smaller than this threshold, although it encompassed materially different magnitudes of benefit. The threshold should be understood as an interpretive benchmark rather than a validated patient-level minimal important difference. The prediction interval was considered under inconsistency and was not counted again under imprecision. Publication bias was not downgraded, although this judgment remained uncertain because the available diagnostics had limited power with only 15 heterogeneous trials. The overall certainty of evidence was very low.

## 4. Discussion

### 4.1. Principal Findings

This review included 17 eligible randomized studies; 15 independent trials involving 1041 participants contributed to the primary synthesis. The average effect favored exercise (*g* = −0.75, 95% CI −1.18 to −0.32), but the prediction interval crossed the null and the magnitude of the pooled effect was sensitive to two influential trials. Excluding Rehman et al. [34] and Wang et al. [31] attenuated the pooled effect to *g* = −0.49 and reduced I^2^ from 87.1% to approximately 0%. The pooled estimates therefore indicated a favorable average direction but not a uniform or precisely transferable effect. Certainty was very low after downgrading for risk of bias, inconsistency, and indirectness; 16 trials had some concerns and one was judged to be at high risk of bias, with none at overall low risk.

### 4.2. Evidence Evolution

Evidence concerning exercise and depressive symptoms in adults with lung cancer has evolved from feasibility-focused reviews to broader quantitative syntheses. Earlier reviews suggested possible psychological benefit but emphasized small trial numbers, heterogeneous interventions, and inconsistent reporting of depression outcomes [12,14,45].

Subsequent meta-analyses reported beneficial pooled effects, including a standardized mean difference of −0.60 across nine randomized trials in Lu et al. [46] and an effect of −0.55 for depressive symptoms in the broader psychological-distress synthesis by Tadsuan et al. [47]. In contrast, Hu et al. [48], which focused on advanced lung cancer, did not identify a statistically significant pooled effect on depressive symptoms. Differences in disease stage, treatment phase, eligibility criteria, intervention composition, outcome selection, and analytical methods likely account for part of this variation.

The present review extends earlier syntheses by retaining one independent primary effect per trial and reporting the prediction interval, influence analyses, risk of bias, and certainty alongside the pooled mean. These analyses show that a favorable average estimate coexists with substantial variation in magnitude and transferability. Evidence from other cancer populations and major depressive disorder is supportive but cannot be directly extrapolated to lung cancer [8,9,10].

### 4.3. Understanding Heterogeneity

The two influential trials differed from most of the dataset. Rehman et al. [34] enrolled 40 patients receiving chemotherapy and compared pulmonary rehabilitation plus aerobic training with pulmonary rehabilitation alone over four weeks. Baseline HADS-D means were 12.75 and 13.02; directly reported post-test means were 4.10 and 9.42 (g = −2.89). Wang et al. [31] enrolled a perioperative stage 0–III surgical population in a three-arm trial. The primary comparison combined PERMA plus loaded deep breathing and loaded deep breathing alone against routine care; discharge HADS-D medians were converted and active arms combined (g = −2.17). Both were rated as having some concerns, but Rehman had limited randomization and analysis reporting, whereas Wang used a per-protocol population, had losses, and required conversion and arm combination. Their extreme estimates may reflect baseline burden, short assessment, small samples, intervention contrast, and analytical assumptions.

The depression data from both trials were rechecked against the original reports, including group sizes, HADS-D scale units, and baseline and post-intervention summary statistics; no transcription or unit errors were identified. Baseline HADS-D values were closely balanced between groups in Rehman et al. (12.75 ± 2.20 vs. 13.02 ± 2.00) and across the three arms in Wang et al. (medians 4 [IQR 3–4.25], 3 [IQR 3–4], and 4 [IQR 3–4]), making baseline imbalance an unlikely explanation for the extreme effects. Rehman’s estimate was calculated from directly reported post-test means and SDs, whereas Wang’s estimate required conversion of discharge medians and combination of the two active arms. The large standardized effects therefore arose principally from substantial short-term between-group separation relative to the reported or derived within-group dispersion. Small sample sizes reduced precision, while the conversion and arm-combination procedures used for Wang et al. introduced additional analytical uncertainty.

Broad subgroup categories did not explain heterogeneity. Baseline depression means were available for 12 quantitative trials, and no study selected participants because of clinically elevated depression. Rehman et al. [34] was the only trial whose group means were clearly above a commonly used HADS-D screening range, which does not establish major depression. An exploratory baseline-severity meta-regression suggested larger effects with greater baseline burden, but it was ecological, based on 12 trials, and sensitive to Rehman et al.; it cannot separate treatment from prevention effects at patient level.

### 4.4. Clinical Interpretation and Sources of Variability

The pooled estimate represents an average effect across clinically and methodologically diverse trials rather than the effect of a single standardized intervention. Participants differed in disease stage, treatment phase, surgical status, functional capacity, and baseline symptom burden. Intervention content, duration, supervision, and delivery also varied, while comparators ranged from usual care and education to self-management and active rehabilitation. Standardization permitted statistical synthesis but did not make these populations, interventions, or contrasts clinically equivalent.

The 95% prediction interval included the null, and exclusion of two influential trials attenuated the pooled effect and removed most of the estimated heterogeneity. These findings limit confidence in the magnitude and transferability of the average effect and do not identify the patients, intervention components, or clinical settings most likely to benefit.

None of the included trials evaluated causal mediators. The review therefore cannot determine whether observed changes arose from exercise-specific effects, nonspecific behavioral support, concurrent changes in physical symptoms, or other components of multicomponent care.

The exercise-dominant sensitivity analysis yielded an average estimate similar to the primary model, indicating that the broader exercise-containing programs did not account for the favorable average direction. However, substantial residual heterogeneity and a prediction interval crossing the null persisted, so this restriction did not establish a uniform exercise-specific effect.

### 4.5. Certainty of Evidence and Clinical Implications

Certainty was very low after downgrading for risk of bias, inconsistency, and indirectness. None of the included trials were judged to be at overall low risk of bias; the interventions and comparator conditions were heterogeneous, and the 95% prediction interval included the possibility of no benefit in a future setting. Confidence in both the magnitude and generalizability of the pooled effect is therefore limited.

The findings do not establish exercise as a treatment for major depressive disorders and do not support a universal exercise prescription for adults with lung cancer. The evidence is also insufficient to recommend a particular modality, intensity, duration, or delivery format for reducing depressive symptoms. Exercise-based interventions may be considered only as potential individualized adjuncts to supportive care when clinically appropriate, feasible, and safe.

Exercise should not replace depression screening, diagnostic assessment, psychotherapy, pharmacological treatment, or specialist psycho-oncology care when these are indicated. Further adequately powered randomized trials are needed to determine whether effects differ according to baseline depressive symptom severity, disease and treatment characteristics, intervention content, and comparator intensity.

### 4.6. Strengths

This review focused specifically on randomized evidence concerning depressive symptoms in adults with lung cancer and maintained one statistically independent primary effect per trial. An explicit hierarchy was applied to outcome, intervention-arm, and time-point selection, and multi-arm trials were handled without double counting shared comparator groups. Published and derived values were distinguished, and median-based conversions, SE-to-SD transformations, combined-arm estimates, and externally derived group-size allocations were examined in robustness analyses.

The primary synthesis used REML random-effects estimation with Hartung–Knapp inference and reported a prediction interval in addition to conventional heterogeneity statistics. Primary, sensitivity, influence, moderator, and small-study-effect analyses were kept analytically distinct. Risk of bias was assessed for the selected depression result using RoB 2, and certainty of evidence was evaluated using GRADE. Together, these procedures enhanced transparency, reproducibility, and the distinction between a favorable average effect and uncertainty in its magnitude and transferability.

### 4.7. Limitations

Only 15 independent randomized trials contributed to the primary meta-analysis, and many had small samples. Although all trials addressed the same broad review question, their populations were not clinically interchangeable. Participants differed in disease stage, treatment phase, surgical status, functional capacity, symptom burden, and baseline depressive symptom severity. The interventions, comparator conditions, depression instruments, and assessment times also varied. Standardized mean differences placed outcomes on a common statistical scale but did not remove these clinical differences. The pooled estimate should therefore be understood as an average across diverse settings rather than as a treatment effect expected in every patient population. This limitation is reinforced by the prediction interval crossing the null and by attenuation of the effect after exclusion of two influential trials.

Disease stage could be classified with sufficient specificity in only 7 of the 15 quantitative trials; categories often overlapped, and several reports combined patients across stages. Oncological treatment likewise ranged from surgery and perioperative care to chemotherapy, chemoradiotherapy, advanced-disease care, and survivorship, with treatment exposure incompletely reported. Formal stage- or treatment-stratified meta-analysis was therefore not performed because the resulting groups would have been sparse, non-exclusive, and vulnerable to misclassification. These variables remain plausible sources of effect modification.

Comparators included usual care, education, self-management, chest physiotherapy, and pulmonary rehabilitation. Active-comparator estimates represent comparative or incremental effects relative to active rehabilitation rather than exercise versus no exercise.

Some effects required median conversion, SD reconstruction, or active-arm combination; raw and derived data are separated; and direct-endpoint sensitivity analysis is reported. One eligible study remained qualitative because conversion was not defensible and another reported only total HADS.

Subgroup and meta-regression analyses were exploratory, involved few studies, and were susceptible to ecological confounding and multiple testing. The age association was sensitive to an influential trial and should not guide clinical decision-making. Small-study-effect tests had limited power; nonsignificant results do not establish the absence of publication bias. The database searches included a depression concept, which may have reduced sensitivity because depressive symptoms were frequently secondary outcomes and may not have been reported in titles, abstracts, or indexing terms. A supplementary search without the depression concept was not conducted. Restriction to English-language publications and the absence of grey-literature and trial-registry searches may also have resulted in relevant evidence being missed.

Nine of the 15 quantitative trials were conducted in China, Hong Kong, or Taiwan. This geographic concentration may limit generalizability to other healthcare systems and cultural contexts, including settings in which intervention delivery, supportive-care pathways, and reporting of depressive symptoms may differ.

### 4.8. Future Research

Future randomized trials should be adequately powered for depressive symptoms and should prospectively specify the depression instrument, primary assessment time point, outcome format, analysis population, and missing-data strategy. Trials should report arm-specific randomized and analyzed sample sizes, means and SDs—or complete distributional summaries for skewed outcomes—at all time points. CONSORT-consistent reporting of intervention adherence, contamination, comparator content, co-interventions, adverse events, and reasons for missing depression assessments is essential.

Eligibility based on clinically meaningful depressive symptoms, or stratification by baseline symptom severity, would help distinguish prevention from treatment effects and reduce floor effects. Trials should also report cancer histology and stage, active treatment, performance status, frailty, concomitant psychological or pharmacological treatment, functional capacity, and cardiopulmonary limitations. Longer-term assessments are required to determine whether benefits persist after intervention completion. Head-to-head, factorial, or component-based designs may help identify which intervention components contribute to benefit. Individual participant data meta-analysis could evaluate patient-level effect modification by baseline depressive symptom severity, disease stage, treatment, age, sex, frailty, performance status, and adherence without relying on ecological study-level comparisons. If a sufficiently connected evidence network emerges, component network meta-analysis may help distinguish the contributions of aerobic, resistance, breathing, mind–body, behavioral, and digital components within multicomponent programs. Future mechanistic and implementation studies should use longitudinal mediator assessment, objective activity or adherence measures where feasible, and standardized definitions of intervention content and adverse events. Development of a core outcome set for psychological outcomes in exercise-oncology trials would improve comparability and reduce selective outcome reporting.

## 5. Conclusions

Exercise-based interventions may reduce depressive symptom scores in some adults with lung cancer, but the magnitude of the average effect was not robust. Exclusion of two influential trials attenuated Hedges’ g from −0.75 to −0.49 and reduced I^2^ from 87.1% to approximately 0%. Together with a prediction interval crossing the null and very-low-certainty evidence, this finding limits the transferability of the pooled estimate across clinical settings. The evidence does not establish efficacy for major depressive disorder or identify an optimal modality, dose, or delivery format. Better-powered trials with clearly characterized populations, comparators, intervention components, and depression-specific outcomes are required.

## Figures and Tables

**Table 1:** Characteristics of studies included in the systematic review.

Study	Sample Size	Age, Years	Female, *n*/N (%)	Cancer Type	Stage	Clinical Phase	Intervention	Comparator	Exercise Category	Duration	Depression Measure/Role	Assessment/Synthesis
Liu et al., 2026 [32]	Randomized 104 (52/52); depression results 77 (38/39)	IG: 60.23 ± 10.56; CG: 61.17 ± 9.57	IG: 26/52 (50.0%); CG: 28/52 (53.8%)	Lung cancer (NSCLC/SCLC)	III–IV	Advanced disease; active or oncological treatment	Hybrid home-based pulmonary rehabilitation	Usual care/education attention control with no prescribed structured exercise	Multicomponent exercise rehabilitation	8 weeks	HADS-D/primary	Week 8; quantitative
Wang et al., 2026 [33]	Randomized 57 (27/30); depression result 50 (25/25)	IG: 54.40 ± 10.87; CG: 58.40 ± 10.44	IG: 14/25 (56.0%); CG: 12/25 (48.0%)	Postoperative NSCLC	Not reported	Postoperative	HIM-guided rehabilitation	Standard care	Digital or telerehabilitation	4 weeks	SDS/secondary	1 month after discharge; quantitative
Wang et al., 2025 [31]	Randomized 102 (34/34/34); depression result 96 (65/31)	IG: 53.32 ± 9.29; CG: 57.84 ± 10.01	IG: 43/65 (66.2%); CG: 25/31 (80.6%)	NSCLC undergoing thoracoscopic lobectomy	I–III (predominantly stage I)	Perioperative	PERMA plus loaded deep breathing; Loaded deep breathing	Routine care	Breathing or respiratory muscle exercise	Admission–discharge (variable)	HADS-D/primary	Discharge; quantitative
Liu et al., 2024 [28]	Randomized 102 (34/34/34); analyzed 99 (33/34/32); narrative synthesis	CIG: 61.06 ± 8.30; MBG: 61.50 ± 9.09; CG: 60.63 ± 8.28	CIG: 18/33 (54.5%); MBG: 16/34 (47.1%); CG: 16/32 (50.0%)	NSCLC undergoing surgery	Carcinoma in situ–III	Perioperative	Mindful breathing plus diary guidance; Mindful breathing	Routine care	Mind–body exercise	Admission–discharge (variable)	HADS-D/secondary	Discharge; narrative synthesis (median and IQR reported)
Rehman et al., 2023 [34]	Randomized 40 (20/20); depression result 40 (20/20)	IG: 48.1 ± 4.0; CG: 48.3 ± 3.8	IG: 9/20 (45.0%); CG: 7/20 (35.0%)	NSCLC	I–II	During chemotherapy	Pulmonary rehabilitation plus aerobic training	Pulmonary rehabilitation	Aerobic exercise added to pulmonary rehabilitation	4	HADS-D/NR	Post-test; quantitative
Lei et al., 2022 [35]	Randomized: 52 (26/26); depression result 37 (19/18)	IG: 56.04 ± 11.67; CG: 58.03 ± 7.71	IG: 9/26 (34.6%); CG: 10/26 (38.5%)	Lung cancer/NSCLC	I–IIIB	Definitive treatment completed more than 1 week previously	Baduanjin plus elastic-band training	Control	Qigong or Baduanjin	8	SDS/primary	Post-intervention; quantitative
Tenconi et al., 2021 [29]	Randomized 140 (70/70); completed 6-month assessment 85 (45/40); narrative synthesis	IG: 66.00 ± 10.61; CG: 67.74 ± 10.84	IG 32/70 (45.7%); CG 22/70 (31.4%)	Clinical stage I–II NSCLC scheduled for resection	Clinical stage I–II	Perioperative	Standard care plus perioperative pulmonary rehabilitation	Standard care	Pulmonary rehabilitation	14–21 days preoperatively plus 8 weeks postoperatively	Total HADS/secondary	6 months; narrative synthesis (HADS-D not separately reported)
Bade et al., 2021 [36]	Randomized 40 (20/20)	IG: 66.55 ± 7.28; CG: 63.20 ± 9.80	IG: 12/20 (60.0%); CG: 18/20 (90.0%)	Stage III/IV NSCLC	IIIA–IV	Active treatment (85%) or post-treatment (15%)	Home-based physical activity	Usual care	Walking	12	PHQ-9/not explicitly designated	Week 12; quantitative (mixed-effects model estimates)
Cheung et al., 2021 [30]	Randomized 30 (19/11)	IG: 61.05 ± 9.76; CG: 58.36 ± 9.32	IG: 8/19 (42.1%); CG: 6/11 (54.5%)	Stage IIIB–IV NSCLC	IIIB–IV	Advanced disease; mixed treatment status	Aerobic exercise; Tai Chi	Self-management control with written exercise guidelines	Aerobic/strengthening exercise and Tai Chi	12	HADS-D/secondary	Post-intervention; quantitative
Quist et al., 2020 [37]	Randomized 216 (108/108)	IG: 65.2 ± 8.2; CG: 63.5 ± 8.7	IG: 55/108 (50.0%); CG: 56/108 (51.9%)	Advanced inoperable lung cancer (NSCLC/SCLC)	Advanced/inoperable	During chemotherapy	Supervised group exercise	Usual care	Combined aerobic and resistance	12	HADS-D/secondary	12 weeks; quantitative (multiple-imputation analysis)
Sui et al., 2020 [38]	Randomized 200 (100/100)	IG: 61.37 ± 11.21; CG: 62.35 ± 9.98	IG: 20/100 (20.0%); CG: 16/100 (16.0%)	NSCLC after surgical resection	I–III	Postoperative; intervention initiated 4–8 weeks after surgery	WeChat education and rehabilitation program	One-time education and rehabilitation guidance plus usual care	Digital or telerehabilitation	52	HADS-D/NR	12 months; quantitative
Wang et al., 2020 [39]	Randomized/result 65 (31/34)	IG: 59 (52–62); CG: 55.5 (46.75–63.25), median (IQR)	IG: 20 (64.5%); CG: 23 (67.6%)	NSCLC receiving surgery	Not reported	Perioperative	Breathing exercises	Routine care	Breathing or respiratory muscle exercise	NR	HADS-D/secondary	Discharge; quantitative
Egegaard et al., 2019 [40]	Randomized 15 (8/7); depression result 12 (7/5)	IG: 64 ± 5.8; CG: 65 ± 4.7	IG: 5/8 (62.5%); CG: 5/7 (71.4%)	Locally advanced NSCLC	locally advanced	During chemoradiotherapy	Aerobic interval training before radiotherapy	Control	Aerobic	7	HADS-D/secondary	7 weeks; quantitative
Cavalheri et al., 2017 [41]	Randomized 17 (9/8)	IG: 66 ± 10; CG: 68 ± 9	IG: 6/9 (66.7%); CG: 6/8 (75.0%)	NSCLC after curative-intent treatment	I–IIIA	6–10 weeks after lobectomy or 4–8 weeks after adjuvant chemotherapy	Supervised combined aerobic and resistance training	Usual activities plus weekly attention-control telephone calls	Combined aerobic and resistance	8	HADS-D/secondary	8 weeks; quantitative
Chen et al., 2015 [42]	Randomized 116 (58/58); depression result 101 (50/51)	IG: 64.76 ± 11.28; CG: 63.57 ± 10.54	IG: 32/58 (55.2%); CG: 30/58 (51.7%)	Lung cancer	I–IV; unknown in 12 participants	Mixed treatment statuses	Home-based walking	Usual care	Walking	12	HADS-D/primary	3 months; quantitative
Morano et al., 2014 [43]	Randomized 24 (12/12)	IG: 65 ± 8; CG: 69 ± 7	IG: 8/12 (66.7%); CG: 7/12(58.3%)	NSCLC awaiting resection with pre-existing inflammatory pulmonary disease	I–IIIA	Preoperative	Pulmonary rehabilitation	Chest physical therapy	Pulmonary rehabilitation	4	HADS-D/NR	1 month; quantitative
Li et al., 2025 [44]	Randomized 47 (25/22); depression result 36 (20/16)	IG: 49.50 ± 11.66; CG: 53.50 ± 10.71	IG: 19/22 (86.4%); CG: 12/18 (66.7%)	Early-stage NSCLC survivors	early stage	Survivorship	Digital cardiac telerehabilitation	Usual care	Digital or telerehabilitation	20	HADS-D/secondary	5 months; quantitative

Notes: Values are presented as mean ± SD unless otherwise indicated. Duration is reported in weeks unless otherwise specified. Sample-size entries distinguish randomized, baseline-demographic, analyzed, outcome-specific, and synthesis populations, as applicable. Female participants are reported as *n*/N (%) using the denominator reported for baseline characteristics, which may differ from the corresponding depression-analysis population. For Wang et al. (2025) and Cheung et al. (2021) [30,31], eligible active intervention arms were pooled for quantitative synthesis; pooled age, SD, and female counts are presented. For Wang et al. (2026) and Li et al. (2025) [33,44], baseline age and sex values refer to study completers included in the published baseline tables. Rehman et al. (2023) and Morano et al. (2014) [34,43] used active rehabilitation comparators; their effect estimates therefore represent comparative or incremental effects relative to active rehabilitation rather than exercise versus no exercise. Abbreviations: CG, comparator group; CIG, combined intervention group; HADS, Hospital Anxiety and Depression Scale; HADS-D, Hospital Anxiety and Depression Scale–Depression subscale; HIM, Hospital–Internet–Mobile; IG, intervention group; IQR, interquartile range; MBG, mindful breathing group; NR, not reported; NSCLC, non-small cell lung cancer; PERMA, Positive Emotion, Engagement, Relationships, Meaning, and Accomplishment; PHQ-9, Patient Health Questionnaire-9; SCLC, small cell lung cancer; SD, standard deviation; SDS, Self-Rating Depression Scale.

**Table 2:** GRADE certainty of evidence for depressive symptoms.

Certainty Assessment							№ of Patients			Certainty	Importance
№ of Studies	Study Design	Risk of Bias	Inconsistency	Indirectness	Imprecision	Other Considerations	Exercise	Control	Standardized Effect (95% CI)		
15	Randomized controlled trials	Serious ^a^	Serious ^b^	Serious ^c^	Not serious ^d^	Publication bias not strongly suspected ^e^	543	498	Hedges’ g = −0.75 (95% CI −1.18 to −0.32)	⨁◯◯◯ Very low	Important

Notes: ^a^ Downgraded by one level for risk of bias because no trial was judged to be at overall low risk and one was judged to be at high risk. ^b^ Downgraded by one level for serious inconsistency because I^2^ was 87.1% and the 95% prediction interval crossed the null. ^c^ Downgraded by one level for serious indirectness because participants were not selected for clinically elevated depression, depression was commonly a secondary outcome, interventions and comparators varied, and settings spanned the care continuum. ^d^ No separate downgrade for imprecision was applied because the confidence interval excluded both the null and an interpretive small-effect threshold of |g| = 0.20, although it permitted materially different magnitudes of benefit. This threshold is not a validated patient-level minimal important difference. ^e^ Publication bias was not downgraded because Egger’s regression, rank correlation, and trim-and-fill did not provide clear evidence of small-study effects; however, these diagnostics had limited power because only 15 heterogeneous trials were available. Abbreviations: CI, confidence interval; GRADE, Grading of Recommendations Assessment, Development and Evaluation; I^2^, percentage of variability attributable to between-study inconsistency.

## Data Availability

The study-level extraction data, analysis-ready dataset, effect-size calculations, data-conversion and multi-arm combination records, RoB 2 assessments, GRADE evidence profile, and R code used for the primary, sensitivity, subgroup, meta-regression, and small-study-effect analyses are publicly available in the OSF project associated with the registration (VUC87): https://osf.io/eyp5v/ (accessed on 17 July 2026). Database-specific search strategies are provided in Supplementary Materials Table S1.

## Supplementary Materials

The following supporting information can be downloaded at: https://www.mdpi.com/article/10.3390/medicina62091805/s1, Table S1. Search strategies used across electronic databases.
